# Supplementary material for: Episiotomies and obstetric anal sphincter injuries following a restrictive episiotomy policy in France: An analysis of the 2010, 2016, and 2021 National Perinatal Surveys
Source: PLoS Med. 2025 Jan 14;22(1):e1004501. doi: 10.1371/journal.pmed.1004501 (PMC11731868; doi:10.1371/journal.pmed.1004501)
Supplement: S1 Table — (DOCX) [file pmed.1004501.s002.docx]

S1 Table: Variations in the prevalence of episiotomy in France between 2010 and 2021 according to the classification for episiotomy practices (Crude Risk Ratio).

| Groups | 2010 | | 2016 | | | 2021 | | |
| --- | --- | --- | --- | --- | --- | --- | --- | --- |
|  | n / N | Episiotomy  % (95% CI) | n / N | Episiotomy  % (95% CI) | cRR [95% CI] | n / N | Episiotomy  % (95% CI) | cRR [95% CI] |
| 1-Nulliparous women, singleton, cephalic, at term, non-instrumental delivery | 878 / 2 479 | 35.4  (33.5-37.3) | 674 / 2 688 | 25.1  (23.4-26.8) | 0.71 [0.65-0.77] | 228 / 2 443 | 9.3  (8.2-10.6) | 0.26 [0.23-0.30] |
| 2a-Nulliparous women, singleton, cephalic, at term, forceps delivery | 300 / 355 | 84.5  (80.3-87.9) | 254 / 313 | 81.2  (76.4-85.1) | 0.96 [0.90-1.03] | 139 / 248 | 56.0  (49.8-62.1) | 0.66 [0.59-0.75] |
| 2b-Nulliparous women, singleton, cephalic, at term, spatula delivery | 190 / 241 | 78.8  (73.2-83.6) | 178 / 253 | 70.4  (64.4-75.7) | 0.89 [0.80-0.99] | 64/ 202 | 31.7  (25.6-38.5) | 0.40 [0.32-0.50] |
| 2c-Nulliparous women, singleton, cephalic, at term, vacuum delivery | 252 / 449 | 56.1  (51.4-60.8) | 241 / 539 | 44.7  (40.5-49.0) | 0.80 [0.70-0.90] | 163 / 635 | 25.7  (22.3-29.3) | 0.46 [0.39-0.53] |
| 3-Multiparous women, singleton, cephalic, at term, non-instrumental delivery | 694 / 5 764 | 12.0  (11.2-12.9) | 399 / 5 161 | 7.7  (7.0-8.5) | 0.64 [0.57-0.72] | 117 / 5 036 | 2.3  (1.9-2.8) | 0.19 [0.16-0.23] |
| 4a-Multiparous women, singleton, cephalic, at term, forceps delivery | 76 / 109 | 69.7  (60.3-77.7) | 50 / 90 | 55.6  (50.0-65.6) | 0.80 [0.64-1.00] | 13 / 47 | 27.7  (16.5-42.6) | 0.40 [0.25-0.64] |
| 4b-Multiparous women, singleton, cephalic, at term, spatula delivery | 59 / 84 | 70.2  (59 .4-79.2) | 37 / 72 | 51.4  (39.7-62.9) | 0.73 [0.56-0.95] | 11 / 63 | 17.5  (9.7-29.2) | 0.25 [0.14-0.43] |
| 4c-Multiparous women, singleton, cephalic, at term, vacuum delivery | 52 / 179 | 29.1  (22.5-36.3) | 57 / 188 | 30.3  (23.8-37.4) | 1.04 [0.76-1.43] | 22 / 235 | 9.4  (6.0-13.8) | 0.32 [0.20-0.51] |
| 5- Singleton, cephalic, < 37 WG | 79 / 422 | 18.7  (15.1-22.8) | 61 / 434 | 14.1  (10.9-17.7) | 0.75 [0.55-1.02] | 18 / 379 | 4.7  (2.8-7.4) | 0.25 [0.15-0.42] |
| 6- Singleton breech pregnancy | 39 / 73 | 53.4  (41.4-65.2) | 21 / 60 | 35.0  (23.1-48.4) | 0.66 [0.44-0.98] | 11 / 56 | 19.6  (10.2-32.4) | 0.37 [0.21-0.65] |
| 7- Multiple pregnancy | 45 / 172 | 26.2  (19.8-33.4) | 40 / 197 | 20.3  (14.9-26.6) | 0.78 [0.53-1.13] | 1 / 84 | 1.2  (0.0 – 6.5) | 0.05 [0.01-0.33] |
| Total | 2 664 / 10 327 | 25.8  (25.0-26.7) | 2 012 / 9 995 | 20.1  (19.3-20.9) | 0.78 [0.74-0.82] | 787 / 9 428 | 8.3  (7.8-8.9) | 0.32 [0.30-0.35] |

WG: weeks of gestation cRR: crude risk ratio. The reference year is 2010 for all analyses.
