# Supplementary material for: Episiotomies and obstetric anal sphincter injuries following a restrictive episiotomy policy in France: An analysis of the 2010, 2016, and 2021 National Perinatal Surveys
Source: PLoS Med. 2025 Jan 14;22(1):e1004501. doi: 10.1371/journal.pmed.1004501 (PMC11731868; doi:10.1371/journal.pmed.1004501)
Supplement: S3 Table — (DOCX) [file pmed.1004501.s004.docx]

**S3 Table:** Variations in the prevalence of episiotomy in France between 2010 and 2021 according to the status of maternity unit

| Maternity unit status | 2010* | | 2016 | | | 2021 | | | P- Trend** |
| --- | --- | --- | --- | --- | --- | --- | --- | --- | --- |
|  | n / N | Episiotomy  % (95% CI) | n / N | Episiotomy  % (95% CI) | aRR ^$^ [95% CI] | n / N | Episiotomy  % (95% CI) | aRR ^$^  [95% CI] |  |
| University hospitals | 439 / 1 784 | 24.6 (22.6-26.7) | 402 / 1 924 | 20.9 (19.1-22.8) | 0.84 [0.75-0.94] | 136 / 1 900 | 7.2 (6.0-8.4) | 0.30 [0.26-0.36] | <0.001 |
| Public hospitals | 1 368 / 5 698 | 24.0 (22.9-25.1) | 1 090 / 5 813 | 18.8 (17.8-19.8) | 0.75 [0.70-0.80] | 485 / 5 540 | 8.8 (8.0-9.5) | 0.34 [0.31-0.37] | <0.001 |
| Private hospitals | 857 / 2 845 | 30.1 (28.4-31.8) | 519 / 2 257 | 23.0 (21.3-24.8) | 0.79 [0.72-0.86] | 166/1 988 | 8.4 (7.2-9.7) | 0.30 [0.25-0.35] | <0.001 |
| Total | 2 663 / 10 318 | 25.8 (25.0-26.7) | 2 011 / 9 990 | 20.1 (19.3-20.9) | 0.78 [0.75-0.82] | 787 / 9 421 | 8.3 (7.8-8.9) | 0.33 [0.30-0.35] | <0.001 |

aRR: Adjusted risk ratios

* Reference. ^**^ Overall trend test across 2010 to 2021 using Cochran-Armitage test

^$^ Adjusted risk ratios obtained after multiple imputation from Poisson regression models with robust variance estimation, adjusted for maternal age, body mass index, country of birth, antenatal classes, suspicion of fetal macrosomia, neuro-axial analgesia during labor, professional who attended the delivery
