# Supplementary material for: Episiotomies and obstetric anal sphincter injuries following a restrictive episiotomy policy in France: An analysis of the 2010, 2016, and 2021 National Perinatal Surveys
Source: PLoS Med. 2025 Jan 14;22(1):e1004501. doi: 10.1371/journal.pmed.1004501 (PMC11731868; doi:10.1371/journal.pmed.1004501)
Supplement: S4 Table — (DOCX) [file pmed.1004501.s005.docx]

| Groups | 2010 | | 2016 | | | 2021 | | |
| --- | --- | --- | --- | --- | --- | --- | --- | --- |
|  | n / N | OASI % (95% CI) | n / N | OASI % (95% CI) | cRR [95% CI] | n / N | OASI % (95% CI) | cRR [95% CI] |
| \| 1-Nulliparous women, singleton, cephalic, at term, non-instrumental delivery \| \| --- \| | 23 / 2 449 | 0.9  (0.6-1.6) | 30 / 2 610 | 1.1  (0.8-1.6) | 1.22 [0.71-2.09] | 27 /2 429 | 1.1  (0.7-1.7) | 1.19 [0.68-2.07] |
| 2a-Nulliparous women, singleton, cephalic, at term, forceps delivery | 11 / 342 | 3.2  (1.8-5.7) | 12 / 290 | 4.1  (2.4-7.2) | 1.27 [0.56-2.87] | 14 / 244 | 5.7  (3.4-9.5) | 1.80 [0.83-3.93] |
| 2b-Nulliparous women, singleton, cephalic, at term, spatula delivery | 6 / 233 | 2.6  (1.2-5.6) | 12 / 220 | 5.5  (3.1-9.4) | 2.05 [0.76-5.51] | 19 / 197 | 9.6  (6.2-14.7) | 3.68 [1.48-9.13] |
| 2c-Nulliparous women, singleton, cephalic, at term, vacuum delivery | 10 / 446 | 2.2  (1.1-4.1) | 7 / 508 | 1.4  (0.6-2.8) | 0.61 [0.24-1.59] | 11 / 625 | 1.8  (0.9-3.1) | 0.78 [0.33-1.82] |
| 3-Multiparous women, singleton, cephalic, at term, non-instrumental delivery | 19/ 728 | 0.3  (0.2-0.5) | 10 / 5 119 | 0.2  (0.1 -0.4) | 0.59 [0.27-1.28] | 16 / 5 020 | 0.3  (0.2-0.5) | 0.96 [0.59-1.87] |
| 4a-Multiparous women, singleton, cephalic, at term, forceps/spatula delivery | 2 / 190 | 1.1  (0.1-3.8) | 2 / 151 | 1.3  (0.2 -4.7) | NC | 5 / 110 | 4.5  (2.5-10.3) | NC |
| 4a-Multiparous women, singleton, cephalic, at term, forceps delivery | 1 / 107 | NC | 1 / 87 | NC | NC | 3 / 47 | NC | NC |
| 4b-Multiparous women, singleton, cephalic, at term, spatula delivery | 1 / 83 | NC | 1 / 64 | NC | NC | 2 / 63 | NC | NC |
| 4c-Multiparous women, singleton, cephalic, at term, vacuum delivery | 1 / 177 | 0.6  (0.0-3.1) | 2 / 182 | 1.1  (0.1-3.9) | NC | 4 / 231 | 1.7  (0.5-4.4) | NC |
| 5- Singleton, cephalic, < 37 WG | 0 / 420 | 0.0  (0.0-0.9) | 4 / 427 | 0.9  (0.3-2.4) | NC | 0 / 377 | 0.0  (0.0-1.0) | NC |
| 6- Singleton breech pregnancy | 0 / 71 | 0.0  (0.0-5.1) | 1 / 55 | 1.8  (0.0-9.7) | NC | 0 / 56 | 0.0  (0.0-6.4) | NC |
| 7- Multiple pregnancy | 2 / 170 | 1.1  (0.1-4.2) | 3 / 193 | 1.6  (0.3-4.5) | NC | 1 / 83 | 1.2  (0.0-6.5) | NC |
| Total | 74 / 10 226 | 0.7  (0.6-0.9) | 83 / 9 755 | 0.9  (0.7-1.1) | 1.20 [0.87-1.64] | 97 / 9 372 | 1.0  (0.8-1.3) | 1.43 [1.05-1.93] |

**S4 Table:** Variations in the prevalence of OASI in France between 2010 and 2021 according to the classification for episiotomy practices (Crude Risk Ratio).

WG: weeks of gestation cRR: crude risk ratio. The reference year is 2010.
