# Supplementary material for: Episiotomies and obstetric anal sphincter injuries following a restrictive episiotomy policy in France: An analysis of the 2010, 2016, and 2021 National Perinatal Surveys
Source: PLoS Med. 2025 Jan 14;22(1):e1004501. doi: 10.1371/journal.pmed.1004501 (PMC11731868; doi:10.1371/journal.pmed.1004501)
Supplement: S5 Table — (DOCX) [file pmed.1004501.s006.docx]

**S5 Table:** Variations in the relative size of each group, the contribution of episiotomy and OASI prevalence according to the classification for episiotomy practices

| Groups | Relative size of each group  % [95% CI] | | | Contribution of each group  to episiotomy prevalence  % [95% CI] | | | Contribution of each group  to OASI prevalence  % [95% CI] | | |
| --- | --- | --- | --- | --- | --- | --- | --- | --- | --- |
|  | 2010 | 2016 | 2021 | 2010 | 2016 | 2021 | 2010 | 2016 | 2021 |
|  |  |  |  |  |  |  |  |  |  |
| \| 1 - Nulliparous women, singleton, cephalic, at term, non-instrumental delivery \| \| --- \| | 24.0  [23.2 – 24.8] | 26.9  [26.0 – 27.8] | 25.9  [25.0 – 26.8] | 33.0  [31.2 – 34.8] | 33.5  [31.4 – 35.6] | 29.0  [25.8 – 32.2] | 31.1  [20.6 – 41.6] | 36.1  [25.8 – 46.4] | 27.8  [18.9 – 36.7] |
| 2a - Nulliparous women, singleton, cephalic, at term, forceps delivery | 3.5 [3.1 – 3.9] | 3.2 [2.9 ­– 3.5] | 2.6 [2.3 – 2.9] | 11.3 [10.1 – 12.5] | 12.6 [11.1 – 14.1] | 17.7 [15.0 – 20.4] | 14.9 [6.8 – 23.0] | 14.5 [6.9 – 22.1] | 14.4 [7.4 – 21.4] |
| 2b - Nulliparous women, singleton, cephalic, at term, spatula delivery | 2.3 [2.0 – 2.6] | 2.5 [2.2 – 2.8] | 2.2 [1.9 – 2.5] | 7.1 [6.1 – 8.1] | 8.9 [7.7 – 10.1] | 8.1 [6.2 – 10.0] | 8.1 [1.9 – 14.3] | 14.4 [6.8 – 22.0] | 19.6 [11.7 – 27.5] |
| 2c - Nulliparous women, singleton, cephalic, at term, vacuum delivery | 4.3  [3.9 – 4.7] | 5.4  [5.0 – 5.8] | 6.7  [6.2 – 7.2] | 9.5  [8.4 – 10.6] | 12.0  [10.6 – 13.4] | 20.7  [17.9 – 23.5] | 13.5  [5.7 – 21.3] | 8.4  [2.4 – 14.4] | 11.3  [5.0 – 17.6] |
| 3 - Multiparous women, singleton, cephalic, at term, non-instrumental delivery | 55.8  [54.8 – 56.8] | 51.6  [50.6 – 52.6] | 53.4  [52.4 – 54.4] | 26.1  [24.4 – 27.8] | 19.8  [18.1 – 21.5] | 14.9  [12.4 – 17.4] | 25.7  [15.7 – 35.7] | 12.1  [5.1 – 19.1] | 16.5  [9.1 – 23.9] |
| 4a - Multiparous women, singleton, cephalic, at term, forceps/spatula delivery | 1.1 [0.9 – 1.3] | 0.9 [0.7 – 1.1] | 0.5 [0.4 – 0.6] | 2.9 [2.3 – 3.5] | 2.5 [1.8 – 3.2] | 1.7 [0.8 – 2.6] | 1.4 [0 – 4.1] | 1.2 [0 – 3.5] | 3.1 [0 – 6.5] |
| 4b - Multiparous women, singleton, cephalic, at term, spatula delivery | 0.8 [0.6 – 1.0] | 0.7 [0.5 – 0.9] | 0.7 [0.5 – 0.9] | 2.2 [1.6 – 2.8] | 1.8 [1.2 – 2.4] | 1.4 [0.6 – 2.2] | 1.3 [0 – 3.9] | 1.2 [0 – 3.5] | 2.1 [0 – 5.0] |
| 4c - Multiparous women, singleton, cephalic, at term, vacuum delivery | 1.7  [1.5 – 1.9] | 1.9  [1.6 – 2.2] | 2.5  [2.2 – 2.8] | 2.0  [1.5 – 2.5] | 2.8  [2.1 – 3.5] | 2.8  [1.6 – 4.0] | 1.3  [0 – 3.9] | 2.4  [0 – 5.7] | 4.1  [0.2 – 8.0] |
| 5 - Singleton, cephalic, < 37 WG | 4.1  [3.7 -4.5] | 4.3  [3.9 – 4.7] | 4.0  [3.6 – 4.4] | 3.0  [2.4 – 3.6] | 3.0  [2.3 – 3.7] | 2.3  [1.3 – 3.3] | 0 | 4.8  [0.2 – 9.4] | 0 |
| 6 - Singleton breech pregnancy | 0.7  [0.5 – 0.9] | 0.6  [0.4 – 0.8] | 0.6  [0.4 – 0.8] | 1.5  [1.0 – 2.0] | 1.0  [0.6 – 1.4] | 1.4  [0.6 – 2.2] | 0 | 1.2  [0 – 3.5] | 0 |
| 7 - Multiple pregnancy | 1.7  [1.5 – 1.9] | 2.0  [1.7 – 2.3] | 0.9  [0.7 – 1.1] | 1.7  [1.2 – 2.2] | 2.0  [1.4 – 2.6] | 0.1  [0 – 0.3] | 2.7  [0 – 6.4] | 3.7  [0 – 7.8] | 1.1  [0 – 3.2] |
| Total | 100 | 100 | 100 | 100 | 100 | 100 | 100 | 100 | 100 |
