# Supplementary material for: Episiotomies and obstetric anal sphincter injuries following a restrictive episiotomy policy in France: An analysis of the 2010, 2016, and 2021 National Perinatal Surveys
Source: PLoS Med. 2025 Jan 14;22(1):e1004501. doi: 10.1371/journal.pmed.1004501 (PMC11731868; doi:10.1371/journal.pmed.1004501)
Supplement: Attachment — Submitted filename: Response_Editor_Reviewers_episio_D-24-01340R1.docx [file pmed.1004501.s007.docx]

Dear Dr DESPLANCHES,

Many thanks for submitting your manuscript "IMPLEMENTATION OF A RESTRICTIVE EPISIOTOMY POLICY: RESULTS FROM THE FRENCH NATIONAL PERINATAL SURVEYS IN 2010, 2016 AND 2021" (PMEDICINE-D-24-01340R1) to PLOS Medicine.
After discussing the paper with the editorial team and an academic editor with relevant expertise, I'm pleased to invite you to revise the paper in response to the reviewers' comments. We plan to send the revised paper to some or all of the original reviewers, and we cannot provide any guarantees at this stage regarding publication.

When you upload your revision, please include a point-by-point response that addresses all of the reviewer and editorial points, indicating the changes made in the manuscript and either an excerpt of the revised text or the location (eg: page and line number) where each change can be found. Please also be sure to check the general editorial comments at the end of this letter and include these in your point-by-point response. When you resubmit your paper, please include a clean version of the paper as the main article file and a version with changes tracked as a marked-up manuscript. It may also be helpful to check the guidelines for revised papers at http://journals.plos.org/plosmedicine/s/revising-your-manuscript for any that apply to your paper.

We ask that you submit your revision by Jul 12 2024 11:59PM. However, if this deadline is not feasible, please contact me by email, and we can discuss a suitable alternative.

Don't hesitate to contact me directly with any questions ([kjanin@plos.org](mailto:kjanin@plos.org)).

Dear Editors,

We thank the Editors and Reviewers for taking the time to review our work and for providing guidance to substantially improve the content and presentation of our manuscript. We have modified the article accordingly to your requests. You will find every modification in the text using track changes, and the pages are noted in the answer for every point below. Please note that the line numbers reflect the manuscript with “no markup”. We hope we have met your requirements to improve this paper.

-----------------------------------------------------------
**Comments from the academic editor:**

**I suggest that to accommodate a broader audience an explanation of restrictive episiotomy and the context of its introduction of France would be beneficial.**

We thank the Editor for this suggestion that will improve the comprehension of the French context. We added the following sentence in the introduction section (page 5 lines 17 and page 6, lines 2):

*“In response to high rates of episiotomies in France compared to international data (5), (71% in nulliparous women in 2003)(17), a French representative association of service users in perinatal care convinced authorities and the French National College of Gynecologists and Obstetricians (CNGOF) to draw up recommendations for clinical practice on episiotomy in 2004. One year later, the CNGOF published guidelines recommending a restrictive episiotomy policy aiming for less than 30% of deliveries with a mediolateral episiotomy (8). This restrictive episiotomy policy has been reiterated in all sets of guidelines associated with vaginal delivery, until the revision of the guideline for the perineal prevention and protection in obstetrics published in 2018 (8, 12, 18-21)”.*

**In order to make these results generalisable to the world, a more detailed discussion about practices in France would be advisable.**

We thank the Editors for highlighting this point. A more detailed discussion about practices in France has been included in this new version of the manuscript. Specifically, we noted that, unlike in other countries, the French guidelines recommend restricted use of mediolateral episiotomy during vaginal delivery, including instrumental delivery. This point has been added to the discussion, please see page 22 lines 4-5:

*“Successive national guidelines promoting a restrictive policy on episiotomy in all childbirth contexts, even for instrumental deliveries”.*

And page 22 lines 5-6

*“Contrary to other international guidelines (10, 11), the 2018 French guidelines recommended a restricted use of episiotomy during vaginal delivery, including instrumental delivery (12)”.*

Additionally, we discussed the fact that vacuum instruments are increasingly used as a first-line protective measure, while forceps and spatulas are used less frequently because of the higher risk of OASI. This point has been added to the discussion, please see page 27, lines 19-22.

*“First, we observed a decrease in the use of forceps and spatula in favor of vacuum delivery during the studied period, which is in line with French guidelines recommending the use of vacuum extraction as a first-line protection against OASI (12)”.*

We also emphasized that prevention and protection of the women's pelvic floor, a recognized method for reducing the risk of OASI *(Okeahialam NA, et al. The prevention of perineal trauma during vaginal birth. Am J Obstet Gynecol. 2023. Published online August 11. DOI: 10.1016/j.ajog.2022.06.021),* is widely practiced in France and may partly explain the lower OASI rates observed *(Riethmuller D, et al. Fetal expulsion: which interventions for perineal prevention? CNGOF perineal prevention and protection in obstetrics guidelines. Gynecol Obstet Fertil Senol. 2018;46(12):937–47).*

This point has been added to the discussion, please see page 22, lines 9-11

*“Manual perineal protection which is known to reduce the risk of OASI (36) is almost systematically performed in France (37) may partly explain the low prevalence of OASI”.*

More generally, the discussion has been widely revised thank to reviewer’s comments.

**Comments from the reviewers:**

**Reviewer #1: Implementation of a Restrictive Episiotomy Policy: Results from the French National Perinatal Surveys in 2010, 2016 and 2021**

**The authors addressed a challenging issue in the field of women's health - decreasing the prevalence of episiotomy with no increase in prevalence of obstetric anal sphincter injury (OASI). The authors assessed the prevalence of both episiotomy and OASI, after the implementation of a restrictive episiotomy policy. Results show significant reduction in prevalence of episiotomy with no significant increase in OASI. Findings can give insights in this challenging issue, giving room to selective episiotomy for women with specific characteristics and also to continuous quality improvement in this field.**

**- A nationwide sample covering three time periods and standardized procedures for data collection were used.**

**- Authors sought ethical approval.**

**- Abstract conveys the main results of the study.**

**- Statistical approach is appropriate**

**- Authors cited recent appropriate research and guidelines**

We thank the reviewer for these positive comments.

**Few points deserve attention and should be explained in more detail. The following are the comments, questions and suggestions:**

**Abstract
The authors stated: "The significant reduction in the prevalence of episiotomy in France has not been followed by an increase in OASI, except in nulliparous women giving birth by forceps/spatula (Group 2a)." Due to the small number of events, there are results for only 4 groups/subgroups (out of all 9 groups /subgroups). This should appear in a clear way in the conclusion section.**

We thank the reviewer for this comment that will enrich the revised version of the manuscript. We agree that the conclusion should be more precise on this point. The analysis of the variation of OASI included 91.5% of women of the whole study population from the four main groups of women. For a better understanding, we now provide this information to the reader.

Consequently, we modified the abstract of the new version of the manuscript as below (please see page 3 and 4):

*“The main limitations of this study include the failure to take into account some potential confounding factors and the inability to analyze some groups of the studied population (8.5% of the sample) because of the very small number of events in these groups”.*

*“Conclusion: The significant overall reduction in the prevalence of episiotomy in France has not been followed by an overall increase in OASI. Analyses performed in the four main groups of women confirmed the main result, except for nulliparous women giving birth by spatula (Group 2b), for whom the rate of OASI significantly increased”.*

We also added this information in the limits paragraph of the discussion section (please see page 21, lines 5-8), as below:

*“Despite the large size of our population, we were not able to analyze OASI trends in all groups given its low frequency. Indeed, analyses on OASI were performed in the four main groups of women, corresponding to 91.5% of the whole study population (26 851/29 353)”.*

**Methods
1 - For readers out of France, it would be welcome an explanation in brief about how French healthcare system (maternity units) works. For example: are there private healthcare services?**

We thank the reviewer for this suggestion. As a consequence, we added information in the methods section (please see page 6, lines 10-16):

*“In France, 99% of women give birth in maternity units (22). In public maternity units (71% of births in 2021)(23), midwives perform all non-instrumental vaginal deliveries and episiotomies if needed. Instrumental vaginal deliveries are performed by obstetricians who perform episiotomies as well if needed in these cases. In private maternity units, obstetricians carry out all childbirth procedures, including non-instrumental and instrumental vaginal deliveries and episiotomies. Since 2005, French guidelines have recommended mediolateral episiotomy (8). In 2010, 97.5% of episiotomies were mediolateral and 2.5% were median (24).*

**2 - What is the refusal rate for each edition of the ENP?**

We thank the reviewer for this question. We agree, this information was lacking. As suggested, we added this point in the methods section (please see page 7, lines 15-16):

*“Women who refused access to their medical records were excluded, corresponding to 0% in 2010, 0.1% in 2016 and 1% in 2021”.*

**3 - Authors should explain the procedures used for statistical modelling. What are the rules to keep variables in the regression models? All independent variables were forced in the models?**

We thank the Reviewer for this comment. We agree this important information was lacking. As suggested, we added this point in the methods section (please see page 10, lines 1-3):

*“These factors were selected on a priori hypotheses according to the literature, on author’s clinical experiences and on the results of the descriptive analysis.”*

**Results**

**1- In the Table 1: meaning of P-value* doesn´t appear at the bottom of the table.**

As also suggested by the third reviewer, the column corresponding to the calculation of the p-value has been removed from Table 1 in the revised version of the manuscript.

**2 - Authors wrote: "The highest decrease was observed in Group 3 [multiparous women with a singleton in cephalic position at ≥37 weeks of amenorrhea and a spontaneous delivery] (aRR 0.20, 95% CI 0.16-0.24)". According to the Table 2, this is true after excluding group 7.**

We thank the Reviewer for this relevant comment. A correction was made to clarify this point in the revised version of the manuscript (page 13 lines 7-10):

*“**The most significant decreases were observed in Group 3 [multiparous women with a singleton in cephalic position at ≥37 weeks of amenorrhea and a spontaneous delivery] (aRR 0.20, 95% CI 0.16-0.24), and in Group 7 [multiple pregnancy] (aRR 0.06, 95% CI 0.01-0.40)”,*

and in the abstract (please see page 3):

*“This reduction was observed in all groups of the classification (P<0.001), ranging from -33% in Group 2a [nulliparous term cephalic singleton with forceps delivery] to -94% in Group 7 [multiple pregnancy]”.*

**3 - In regards to the S1 Table and S2 Table, I don't understand the information at the bottom: "Adjusted risk ratios obtained after multiple imputation from Poisson regression models with robust variance estimation, adjusted for maternal age, BMI, country of birth, antenatal classes, suspicion of fetal macrosomia, neuro-axial analgesia during labor, professional who attended the delivery**, and maternity unit size and status." I suppose the number of deliveries is an indicator of maternity unit size. Why did authors adjust for maternity unit size if analysis was stratified by this variable? The same for the variable maternity unit status.**

We thank the Reviewer for spotting this error due to a copy-paste mistake in a footnote. We did not adjust and stratify by the same variable. We proceeded to the correction in the footnotes of Tables S1 and S2. (Please see pages 15 and 16).

The word "volume" has been changed by the word “number”, please page 9 lines 14, page 10 lines 7, Table 1 and S1 Table.

**Moreover, if these Tables display results for all groups as a whole, I don't understand why authors wrote at the bottom: "**Professional who attended the delivery was not introduced into the model for the analysis of groups 2a, 2b, 4a and 4b because instrumental delivery can only be used by obstetricians in France."**

We understand the reviewer’s comment. This was a mistake, the sentence has been removed.

**Discussion/Conclusion
Authors should be cautious in regards to their conclusions about variation in prevalence of OASI. They stated: "Analyses using a seven-group classification system showed that the drop in episiotomy use across all groups not followed by a notable increase in OASI, except among nulliparous women delivering with forceps/spatula." However, it is important to say they were not able to analyze OASI trends in all groups due to the small number of events. Indeed, there are results for only 4 groups/subgroups (out of all 9 groups /subgroups). This should appear in a clear way in the conclusion section.**

We thank the reviewer for this comment. As explained for the previous comment of the conclusion of the abstract, we also proceeded to change in the conclusion of the manuscript to clarify this point (please see pages 24, lines 9-19):

*“A restrictive episiotomy policy has been successfully implemented in France, and the drop in episiotomy use between 2011 and 2021 across all groups in our classification was not followed by an overall increase in OASI. Analyses performed in the four main groups of women, representing 91% of the study population, confirmed the main result, with the exception of nulliparous women giving birth by spatula (Group 2b). In this group, the prevalence of episiotomy was reduced by more than half, while the prevalence of OASI tripled”.*

**Reviewer #2: This is a cross-sectional study of vaginal births >21 weeks' gestation in France at three time points 2010, 2016, 2021 with data obtained from a national perinatal survey. They aimed to assess temporal changes in episiotomy and obstetric anal sphincter injury (OASI) across these time points They split the population into seven clinically informative groups, assessed the distribution of relevant characteristics, the rate of episiotomy, and the rate of OASI, in each group across all three time points. They also used multivariable regression to assess adjusted relative changes in episiotomy and OASI rates in 2016 and 2021 vs 2010. Episiotomy decreased in all groups and OASI either decreased or was stable in all groups except for the nulliparous groups with spatula/forceps delivery. In the spatula forceps group, the episiotomy rate decreased by 50% and the OASI rate doubled.
This is a well-written study with some interesting findings. There are some important issues that should be addressed.**

We thank the Reviewer for these positive comments.

**1. The type of episiotomy (median, mediolateral, lateral) is not mentioned or discussed. This is a large omission given the well-established differences in the relationship between type of episiotomy and OASI risk. The type of episiotomy used, the angle of episiotomy (with mediolateral), and the potential for changes in these factors over time are critical to understanding these trends and their relationship with OASI trends. If these data are not available, provide more detail about episiotomy protocols in France (are they uniform?).**

We thank the Reviewer for this comment. We omitted a description of the technique because practices are widely homogeneous in our country. This was a mistake on our part. Since 2005, guidelines have recommended mediolateral episiotomy. In 2010, the national perinatal survey reported that 97.5% of episiotomies were mediolateral and 2.5% were median according to ENP 2010 results.

In the revised version of the manuscript, this information has been included in the new paragraph on French practices included in the methods section (page 6, lines 15-16):

*“Since 2005, French guidelines have recommended mediolateral episiotomy (8). In 2010, 97.5% of episiotomies were mediolateral and 2.5% were median (24)”.*

**2. It is useful to have separated the population into the seven clinically meaningful groups. These are very similar to the Robson 10-group classification for cesarean delivery and seem to be a modification of this system. It may be worthwhile to frame this classification as a modified Robson classification as it is very similar and the Robson classification has international recognition and uptake.**

We thank the reviewer for the opportunity to clarify the methodology behind the creation of the group classification. Both classifications are indeed based on the concept of linking an intervention to a clinical context that justifies the use of that specific intervention. However, a significant difference lies in the fact that the groups were constructed to reflect clinically relevant situations concerning the risk of caesarean section for Robson on one hand and the risk of episiotomy on the other hand. Consequently, different criteria were used for their construction. In addition, the classification published by Desplanches et al *(Desplanches T, Szczepanski E, Cottenet J, Semama D, Quantin C, Sagot P. A novel classification for evaluating episiotomy practices: application to the Burgundy perinatal network. BMC Pregnancy Childbirth. 2019;19(1):300)* was defined by statistical methods such as ascending hierarchical classification. Therefore, this classification is not a modified Robson classification but rather a specific classification designed to study episiotomies and OASIs.

We provided details in the paragraph titled "Strengths" in the discussion section, as seen below (page 20, lines 14-18):

*“The variations in the prevalences of both episiotomy and OASI were studied concomitantly and according to a clinically relevant classification of obstetric contexts specifically built to assess variations in the risk of episiotomy and OASI, providing new knowledge and opening up new perspectives for clinical practice and research”.*

**3. While it is helpful to see these distinct groups it is not clear why births with spatula and forceps are presented together? These are two distinct instruments. Data from France regarding forceps are often not comparable to other contexts because they are aggregated with spatula in this way. Since most other countries do not use spatula, it is difficult to interpret/generalise. Unless one of these instruments are used far more than the other, there should be sufficient numbers to provide stable rates (out of a total of 835, 778, and 596 deliveries in 2010, 2016, 2021, resp.). If one group is far larger than the other (spatula often accounts for >85% of these combined spatula/forceps groups in other publication from France), than this should be clarified. The rates of OASI in these disaggregated groups should also be reported. It is possible the rates and trends of episiotomy and OASI are different in these two groups and it would be important to understand if the aggregated rates are reflective of the experience with either or both instruments. This disaggregation should appear in Table 1 and in all in the other results tables with group 2 being split into 2a - forceps, b-spatula, and c-vacuum and 4a-forceps, 4b-spatula, 4c-vacuum. As the forceps/spatula group have the highest rates of epis and OASI and the inverse association with the temporal trends (decreasing epis, increasing OASI) it is important to focus and be detailed in this group.**

We agree with the reviewer that forceps and spatulas are two distinct instruments. Their frequency of use was very similar with 4.7% in 2010, 4.3% in 2016 and 3.3% in 2021 for forceps delivery, and 3.4% in 2010, 3.5% in 2016 and 3.0% in 2021 for the spatula delivery. This information has been added to the new version of Table 1 (Please see page 12).

Despite a difference in their mode of action *(Christophe Vayssière, et al Instrumental delivery: clinical practice guidelines from the French College of Gynaecologists and Obstetricians,European Journal of Obstetrics & Gynecology and Reproductive Biology, Volume 159, Issue 1, 2011,Pages 43-48, ISSN 0301-2115)*, they are associated with a similar risk of OASI *(Lebraud M, et al. Comparison of maternal and neonatal outcomes after forceps or spatulas-assisted delivery. Eur J Obstet Gynecol Reprod Biol. 2021 Mar;258:126-131. doi: 10.1016/j.ejogrb.2020.12.057. Epub 2020 Dec 31. PMID: 33421809).*

However, we agree with the reviewer that episiotomy practices may have evolved differently for these two instruments, and that these variations may have had an impact on OASI. We have therefore detailed the original classification to take into account the three different types of instrument.

These modifications have been added, please see:

- Box 1: Seven-Group Classification according to Desplanches et al (16). (Page 9).

| **Groups** | **Seven-Group Classification** |
| --- | --- |
| 1 | Nulliparous women with a single cephalic pregnancy at ≥37 weeks of amenorrhea, non-instrumental delivery |
| 2 | Nulliparous women with a single cephalic pregnancy at ≥37 weeks of amenorrhea, instrumental delivery |
|  | **2a forceps delivery** |
|  | **2b spatula delivery** |
|  | **2c vacuum delivery** |
| 3 | Multiparous women with a single cephalic pregnancy at ≥37 weeks of amenorrhea, non-instrumental delivery |
| 4 | Multiparous women with a single cephalic pregnancy at ≥37 weeks of amenorrhea, instrumental delivery |
|  | **4a forceps delivery** |
|  | **4b spatula delivery** |
|  | **4c vacuum delivery** |
| 5 | All women with a single cephalic pregnancy at <37 weeks of amenorrhea |
| 6 | All women with a single breech pregnancy |
| 7 | All women with multiple pregnancy |

- In the methods section. Page 8 lines 17-20:

*“While forceps and spatula were grouped together in this classification, we assessed them separately to better study episiotomy and OASI rates for each instrument. Consequently, we detailed the type of instruments used, i.e. forceps, spatula or vacuum, in groups 2 (2a, 2b, 2c respectively) and 4 (4a, 4b, 4c, respectively)”.*

- We have updated the footnotes of the Tables 2 and 3 (please see page 14 and 18).
- We have also updated the result section (please see page 11 lines 7-10).

*“However, the type of instrument used for delivery changed: the proportion of vacuum deliveries increased from 6.4% to 9.5%, while the proportion of forceps and spatula deliveries decreased from 4.7% to 3.3% and 3.4% to 3.0%, respectively (Table 1)”.*

- The results of this new analysis are presented in abstract (please see page 3):

*“By groups of classification, the prevalence of OASI increased significantly only in Group 2b (nulliparous term cephalic singleton with spatula delivery) (aRR 3.69, 95% CI 1.50-9.09), and non significantly in Group 2a [nulliparous term cephalic singleton with forceps delivery] (aRR 1.78, 95% CI 0.81-3.90)”.*

- New Tables (Table 2, Table 3, and S3 Table). Please see page 14, 18, and 19.

They provide new insights, particularly regarding the risk of OASI in case of forceps and spatula delivery (Please see page 17 lines 4-10).

*“However, analyses using the seven-group classification revealed a significant increase in the prevalence of OASI within Group 2b [Nulliparous women, singleton, cephalic, at term, spatula delivery], from 2.6% (95% CI 1.2-5.6) in 2010 to 9.6% (95% CI 6.2-14.7) in 2021 (aRR 3.69, 95% CI 1.50-9.09). The prevalence of OASI within Group 2a [Nulliparous women, singleton, cephalic, at term, forceps delivery] increased from 3.2% (95% CI 1.8-5.7) to 5.7% (95% CI 3.4-9.5), but this increase was not significant after adjustment (aRR 1.78, 95% CI 0.81-3.90) (Table 3)”.*

This point was added in the discussion section: please see page 22 lines 6-24, and page 23 lines 1-12

*“We found that the steep overall drop in episiotomy rates in France was not followed by a marked increase in OASI. The overall prevalence of OASI increased slightly to around 1% in 2021, but the increase was not significant and the prevalence remains very low compared with many other countries (33). Manual perineal protection which is known to reduce the risk of OASI (36) is almost systematically performed in France (37) may partly explain the low prevalence of OASI. A review of literature has shown conflicting conclusions (38). Some studies have reported a stable prevalence of OASI over time, while others have observed a moderate to substantial increases concurrent to decreasing episiotomy rates (13, 14, 39-41).*

*Our findings are reassuring considering that we did not detect significant increases in the overall prevalence of OASI but trends in the prevalences of episiotomy and OASI should be interpreted by subgroups of women to better account for the diversity of obstetric contexts. Indeed, among nulliparous women with single cephalic delivery at term with forceps (Group 2a) and spatula (Group 2b), the prevalence of OASI doubled or even tripled in case of spatula delivery. Two likely interconnected factors may have contributed to this increase. First, we observed a decrease in the use of forceps and spatula in favor of vacuum delivery during the studied period, which is in line with French guidelines recommending the use of vacuum extraction as a first-line protection against OASI(12). It is therefore possible that forceps and spatula were increasingly used in more complex obstetric situations, leading to an increased risk of OASI in 2021 compared to 2016.* *Secondly, there was a steep reduction in episiotomy prevalence in these two groups, and the rates of episiotomy became low in 2021. We found a statistically significant association only in spatula deliveries, where the reduction in the prevalence of episiotomy was more marked than in forceps deliveries. Nonetheless, given the small number of OASI in forceps deliveries, the results should be considered with caution. Contrary to other international guidelines(10, 11), the 2018 French guidelines recommended a restricted use of episiotomy during vaginal delivery, including instrumental delivery(12). Considering the increased prevalence of OASI observed in our study, and in light of accumulating new evidence from observational studies published after 2018 (36, 39-42) suggesting a protective role of episiotomy against OASI in instrumental deliveries, it may be worth reconsidering the substantial reduction in episiotomy rates in populations at a high risk of OASI and revising indications of episiotomy in specific contexts of instrumental deliveries in the French guideline”.*

**4. Why was onset of labour (spontaneous/induced) not collected? The frequency of induction has certainly changed over this time period in many settings and may be related to the trends observed in this analysis.**

We thank the reviewer for this question. We did not include the onset of labour in analyses for two reasons. First, we wanted to avoid any over adjustment of risk with the introduction in the models of both macrosomia and mode of onset of labour, as they are correlated. Choosing macrosomia seemed more relevant as this characteristics could be more directly clinically linked with the risk of perineal laceration. Second, the rate of induction did not notably change during the study period (22.7 % in 2010, 22.0 % in 2016 and 25.8 % in 2021). This has been detailed in the paragraphs limits of the discussion section (page 21, lines 11-13).

*“The mode of onset of labor was not included in our models to avoid an over-adjustment of risk with macrosomia and because the rate remained stable during the study period (22.7% in 2010, 22.0% in 2016 and 25.8% in 2021) (23)”.*

**5. Do the restrictive episiotomy guidelines in France extend beyond spontaneous vaginal delivery to include instrumental vaginal delivery? This is not consistent with other national guidelines (RCOG, ANZOG), which specify that episiotomy should be considered with instrumental birth, especially with forceps delivery and among nullipara. If so, the appropriateness of the French guideline (and these inconsistencies with the aforementioned guidelines) should be discussed here. Especially in light of accumulating evidence from observational (Okeahialam NA, et al. Mediolateral/lateral episiotomy with operative vaginal delivery and the risk reduction of obstetric anal sphincter injury (OASI): A systematic review and meta-analysis. Int Urogynecol J. 2022;33(6):1393-405) and experimental (Bergendahl S. Vacuum extraction and pelvic floor injury: A randomized controlled trial and cohort studies. Stockholm, Sweden: Karolinska Institutet; 2024) studies showing >50% decreases in OASI with vs without mediolateral/lateral episiotomy. The fact that the rate is decreasing with forceps/spatula/and vacuum should give us pause. What is the evidence driving this decrease in episiotomy among instrumental deliveries? Since these recommendations, observational studies (refs) and experimental studies (Bergendahl S. Vacuum extraction and pelvic floor injury: A randomized controlled trial and cohort studies. Stockholm, Sweden: Karolinska Institutet; 2024) showing the protective role of medio-lateral episiotomy. The fact that the rate decreases with forceps, spatula and suction cup should give us pause for thought. What is the evidence for this reduction in episiotomy among instrumental deliveries?**

We thank the reviewer for this comment. The restrictive guidelines on episiotomy in France extend beyond spontaneous vaginal delivery. This point has been clarified in the discussion please see page 22 lines 4-5:

*“Successive national guidelines promoting a restrictive policy on episiotomy in all childbirth contexts, even for instrumental deliveries”.*

Furthermore, we totally agree with the reviewer that given the results of our study, and in light of the results of new recent studies showing the protective role of mediolateral episiotomy, this should make us think about the restrictive policy led in France since the 2018 recommendations. This point has been clarified in the discussion please see page 22 line 24 and page 23-lines 1-12:

*“Secondly, there was a steep reduction in episiotomy prevalence in these two groups, and the rates of episiotomy became low in 2021. We found a statistically significant association only in spatula deliveries, where the reduction in the prevalence of episiotomy was more marked than in forceps deliveries. Nonetheless, given the small number of OASI in forceps deliveries, the results should be considered with caution. Contrary to other international guidelines(10, 11), the 2018 French guidelines recommended a restricted use of episiotomy during vaginal delivery, including instrumental delivery(12). Considering the increased prevalence of OASI observed in our study, and in light of accumulating new evidence from observational studies published after 2018 (36, 39-42) suggesting a protective role of episiotomy against OASI in instrumental deliveries, it may be worth reconsidering the substantial reduction in episiotomy rates in populations at a high risk of OASI and revising indications of episiotomy in specific contexts of instrumental deliveries in the French guideline”.*

**6. It seems failed attempts of instrumental delivery have been excluded and not accounted for. This should be acknowledged and how this omission might change affect the study's results should be discussed.**

We thank the reviewer for raising this point. We did not report failed attempts of instrumental deliveries because this information was not collected. It would have been useful to assess as it could have explain contexts of higher risk of OASI in some groups. As suggested we added this point in the paragraphs limits of the discussion section as below (page 21, lines 3-5):

*“Information about failed attempts of instrumental delivery was not collected although it would have been useful to investigate as a means of potentially explaining higher rates of OASI in some groups”.*

**7. The <2% rate of OASI in vacuum delivery among nullipara is very low compared to other high-income settings and it is difficult to decipher the reasons for these relatively low rates of OASI with vacuum without further clinical details (length of labour, fetal station, fetal position). As a result, the finding of this analysis showing that decreased episiotomy were seen while rates of OASI were stable, should be interpreted with caution as the generalisability of these results are questionable. This should be further emphasised given the mounting evidence showing lower rates of OASI with episiotomy with vacuum birth, particularly in nullipara.**

We thank the Reviewer for this relevant comment. We agree that this result should be interpreted with caution. A paragraph highlighting this point has been added to the discussion. Please see page 23, lines 13-22.

*“Another important finding is the stable prevalence (2%) of OASI in nulliparous women who delivered a full-term infant in the cephalic position with vacuum (Group 2c), even though episiotomy use was divided by two in this group. The more frequent use of vacuum-assisted delivery may also have influenced the overall prevalence of OASI since vacuum-assisted delivery is associated with a reduced risk of OASI compared with forceps or spatula delivery (37). However, these results should be interpreted with caution due to the low prevalence of OASI in our study compared to the literature (43). Additionally, accumulating evidence from both observational studies (39, 41, 42) and a recent multicenter, open label, randomized controlled trial (44) found a significant reduction in OASI with lateral episiotomy compared to no episiotomy.”*

**One minor comment, consider changing the titles of the tables to remove the word "evolution". These estimates do not present an "evolution" of factors. This is an analysis of three fairly disparate snapshots in time and it is not clear that these trends are linear between these time points.**

Thank you for your comment. As suggested as well by the reviewer #3, we changed the word "evolution" by the word “variations”. Please see page 10 line 10, page 25 line 16, Table 2, S1 Table, S2 Table, Table 3, and S3 Table.

**Reviewer #3: Thanks for the opportunity to read your manuscript. My role is statistical reviewer, so I have focused on the design, data, and analysis that are presented. I have put general comments first, followed by questions relevant to a specific section of the manuscript (with a page/paragraph reference).**

**This study presents changes in rates of episiotomies in women from France, over 2010, 2016, and 2021. Data is from a population-based based survey of all live births with live vaginal delivery in women 18 years or over. Third and fourth degree tears were considered as a secondary variable, and a range of covariates was collected from the participants, the birth, and the site where the birth took place. Changes in rates were considered stratified by a previously established 7-group classification that incorporates parity and delivery mode. Changes in the outcomes were analysed with a C-A test, and a Poisson regression model that included potential confounders of rates of the outcomes. I thought this was a clearly written paper with interesting findings, and apart from a few queries the analysis was appropriate.**

We thank the Reviewer for these positive comments.

**Given the size of the study, any comparison of characteristics between years is likely to yield a small p-value for even a minor difference. I would remove the p-values from the results.**

We thank the reviewer for this comment. As suggested, we have removed the P values from Table 1. The sections on statistical methods and results have been revised accordingly.

**Was there any data available on how many women refused the survey each year?**

We thank the reviewer for this question. We agree, this information was lacking. As suggested, we added this point in the methods section (please see page 7, lines 15-16):

*“Women who refused access to their medical records were excluded, corresponding to 0% in 2010, 0.1% in 2016 and 1% in 2021”.*

**An optional suggestion - the table are fairly large, and you might be able to use some visualisations to describe the data in a more effective way.**

Thank you for this suggestion we considered. Nonetheless, to provide to readers all the necessary information, we preferred to present the results in tabular form.

**P7, Paragraph 3. Should be 'multivariable', not 'multivariate'**

The correction has been made. Please see page 10, line 1.

**P8, Paragraph 1. Should this be 'variation' instead of 'evolution'?**

Thank you for your comment. As suggested as well by reviewer #2, we changed the word "evolution" by the word “variations”. Please see page 10 line 10, page 25 line 16, Table 2, S1 Table, S2 Table, Table 3, and S3 Table.

**P8, Paragraph 2. What criteria was used to decide on 10 imputations?**

According to Ruben’s rules and regular practices in biostatistics we generated a number of independent imputed datasets close to the percentage of missing data. With data missing for 8.3% of women, we decided to generate 10 independent imputed datasets.
