# Supplementary material for: Episiotomies and obstetric anal sphincter injuries following a restrictive episiotomy policy in France: An analysis of the 2010, 2016, and 2021 National Perinatal Surveys
Source: PLoS Med. 2025 Jan 14;22(1):e1004501. doi: 10.1371/journal.pmed.1004501 (PMC11731868; doi:10.1371/journal.pmed.1004501)
Supplement: Attachment — Submitted filename: Response_Editor_Reviewers_episio_D-24-01340R2.docx [file pmed.1004501.s008.docx]

Dear Dr DESPLANCHES,

Many thanks for submitting your manuscript "IMPLEMENTATION OF A RESTRICTIVE EPISIOTOMY POLICY: RESULTS FROM THE FRENCH NATIONAL PERINATAL SURVEYS IN 2010, 2016 AND 2021" (PMEDICINE-D-24-01340R2) to PLOS Medicine. The paper has been re-reviewed by subject experts and a statistician; their comments are included below and can also be accessed here:

After discussing the paper with the editorial team and an academic editor with relevant expertise, I'm pleased to invite you to revise the paper in response to the reviewers' comments. We plan to send the revised paper to some or all of the original reviewers, and we cannot provide any guarantees at this stage regarding publication. When you upload your revision, please include a point-by-point response that addresses all of the reviewer and editorial points, indicating the changes made in the manuscript and either an excerpt of the revised text or the location (eg: page and line number) where each change can be found. Please also be sure to check the general editorial comments at the end of this letter and include these in your point-by-point response. When you resubmit your paper, please include a clean version of the paper as the main article file and a version with changes tracked as a marked-up manuscript.

We ask that you submit your revision by **Sep 17 2024 11:59PM.** However, if this deadline is not feasible, please contact me by email, and we can discuss a suitable alternative.

Don't hesitate to contact me directly with any questions ([kjanin@plos.org](mailto:kjanin@plos.org)). Best regards,
Katrien Katrien Janin, PhD Associate Editor PLOS Medicine kjanin@plos.org

Dear Editor,

We thank the Editors and Reviewers for taking the time to review our work. We have modified the article accordingly to your requests. You will find every modification in the text using track changes, and the pages are noted in the answer for every point below. Please note that the line numbers reflect the manuscript with “no markup”. We hope we have met your requirements to improve this paper.

**Comments from the reviewers:**

**Reviewer #1: Title: IMPLEMENTATION OF A RESTRICTIVE EPISIOTOMY POLICY: RESULTS FROM THE FRENCH NATIONAL PERINATAL SURVEYS IN 2010, 2016 AND 2021 I read the revised manuscript. The authors addressed all my comments and questions. I have no more comments. In my opinion the manuscript should be published.**

We thank the Reviewer 1 for this positive comment.

**Reviewer #2: The authors have done a nice job addressing the Reviewers' comments. The manuscript is improved. Still, in the interest of patient safety, the authors need to be clearer about the distinction between episiotomy practice** **in instrumental vaginal delivery and non-instrumental vaginal delivery and their results in these groups. The mention of these issues in the Discussion is appreciated, but this needs to be better articulated in the Abstract and in the Conclusion.**

We thank Reviewer 2 for their encouraging comments on our work in the first review. We agree with the suggestion that we should better distinguish between episiotomy practices in instrumental vaginal delivery and non-instrumental vaginal delivery, and we have revised the manuscript accordingly.

**Abstract
It is stated that the rate of OASI in nullipara with forceps (Group 2a) increased but that this was not statistically significant. It should be noted that this is most likely due to a type II error. Given the 342 forceps deliveries in 2010 and 244 forceps deliveries in 2021, with an alpha=0.05 and a beta=0.80 (80% power) the smallest detectable difference would be an rate ratio (RR) of 2.90 (even before including confounders in the model). The observed increase in OASI from 3.2% to 5.7% represents a 55% relative increase OASI. While this was not statistically significant due to lack of power, this should be highlighted as a clinically significant increase. This may seem trivial in this unique population with VERY low rates of OASI with forceps delivery, in most places that use forceps delivery often, the rates of OASI among these deliveries are from 8-25%. A 55% increase in these rates would be enormously significant.**

**As a result, the forceps delivery group should not be included in this interpretation. For example, the conclusion of the Abstract states: "Analyses performed in the four main groups of women confirmed the main result, except for nulliparous women giving birth by spatula (Group 2b), for whom the rate of OASI significantly increased." Group 2a also did not confirm the main result - the study simply did not have enough power to support or refute this in group 2a. Group 2c (vacuum deliveries with nullipara) similarly suffers from power issues (a minimum detectable difference RR=2.65). Most readers will get their main take aways from the Abstract and it is important that these nuances are not misrepresented here. The final sentence in the abstract should be specific to spontaneous vaginal deliveries.**

We thank the reviewer for this comment that will enrich the revised version of the manuscript. We agree that the conclusion should be more precise on this point.

Consequently, we modified the abstract of the new version of the manuscript as seen below (please see page 3 and 4):

*“The significant overall reduction in the prevalence of episiotomy in France has not been followed by an overall increase in OASI. However, subgroup analyses revealed a significant rise in OASI among ~~Analyses performed in the four main groups of women confirmed the main result, except for~~ nulliparous women giving birth by spatula (Group 2b), ~~for whom the rate of OASI significantly increased~~ and a clinically relevant but non-significant rise among nulliparous women delivering by forceps (Group 2a), while the prevalence of episiotomy significantly decreased. These results should be interpreted with caution given the low prevalence of OASI in some subgroups. Further research is needed to predict the optimal rate of episiotomy for instrumental deliveries. In hospitals with high episiotomy rates, episiotomy could be safely reduced for spontaneous vaginal deliveries to comply with international guidelines and women's requests”.*

To maintain consistency between the results and the conclusion, we believe it is essential to include the following results in the abstract:

*“By groups of classification, the prevalence of OASI increased significantly in Group 2b [nulliparous term cephalic singleton with spatula delivery] from 2.6% (95% CI 1.2-5.6) to 9.6% (95% CI 6.2-14.7) (aRR 3.69, 95% CI 1.50-9.09), and non significantly in Group 2a [nulliparous term cephalic singleton with forceps delivery] from 3.2% (95% CI 1.8-5.7) to 5.7% (95% CI 3.4-9.5) (aRR 1.78, 95% CI 0.81-3.90)”.*

**Introduction
In lines 7-9 of the introduction, there is no distinction between instrumental and non-instrumental vaginal delivery. International guidelines are consistent in recommending that episiotomy is restrictive with non-instrumental vaginal delivery but not with instrumental vaginal delivery. The UK and Australia have episiotomy rates ~90% with forceps delivery. This distinction is important. Obstetricians who are proficient in forceps delivery in most countries will almost always perform a mediolateral or lateral episiotomy with forceps delivery in nulliparous patients to avoid OASI. Suggesting that episiotomy rates should be decreased in this population is not supported by this study, is contradicted by other studies, and could result in an increase in these severe injuries.**

We thank the reviewer for this relevant suggestion. We have modified the introduction to distinguish episiotomy practice recommendations between instrumental and non-instrumental vaginal deliveries. Please see page 5 lines 7-9 *“Evidence from the literature therefore suggests the benefit of a restrictive practice of episiotomy and international guidelines have been consistent for many years in recommending that episiotomy use should be limited during spontaneous vaginal birth”.*

**Conclusion**

**Again, the first sentence again does not describe the differences in the non-instrumental and instrumental vaginal delivery groups and states that a drop in episiotomy "across all groups" was not followed by an overall increase in OASI. The second sentence also ignores the likely type II error and generalizes the non-instrumental vaginal delivery findings to all groups. There should be an explicit distinction between the confidence in the estimates in the instrumental and non-instrumental vaginal delivery groups.**

We thank the reviewer for the opportunity to clarify our conclusion. We have made the following changes (please see page 24, lines 9-19 and page 25 lines 1-2):

*« A restrictive episiotomy policy has been successfully implemented in France for both spontaneous and instrumental deliveries, and ~~the drop in episiotomy use between 2011 and 2021 across all groups in our classification~~ was not followed by an overall increase in OASI. ~~Analyses performed in the four main groups of women~~**~~, representing 91% of the study population, confirmed the main result, with the exception of~~ However, subgroup analyses revealed a significant increase in OASI in nulliparous women giving birth by spatula (Group 2b). In this group, the prevalence of episiotomy was reduced by more than half, while the prevalence of OASI tripled. A clinically relevant but non-significant increase was observed in the prevalence of OASI in nulliparous women delivering with forceps (group 2a). No increase in OASI was observed in nulliparous women delivering with vacuum. Results in subgroups 2a and 2c should be interpreted with caution given the low prevalence of OASI and potential lack of statistical power. Further studies with a high level of evidence are needed to predict the optimal rate of episiotomy for this group of women at high risk of OASI. Finally, our findings suggest that ~~in the vast majority of obstetric contexts, particularly in hospitals and regions where episiotomy rates remain high,~~ episiotomy use can be safely reduced for spontaneous vaginal deliveries, particularly in hospitals and regions where episiotomy rates remain high, to comply with international guidelines and women's requests ».*

**Minor points**

**Note the test for linear trend is Cochran-Armitage not Cochrane-Armitage.**

Thank you for your comment. A correction has been made. Please see abstract section.

**Pg 5, Line 6 - the references 1-4 do not support the associations stated, for example, there is no association between episiotomy and PPH shown in these four references. Suggest reviewing these refs and citing the original research papers to support your opening statement.**

We have now updated the references as follows: *“it is also associated with severe maternal complications such as postpartum hemorrhage (1,2), urinary retention (3), infection (4,5), ~~perineal pain,~~ dyspareunia (6), anxiety (7), and post-traumatic stress disorder (8) ~~(1-4)~~”.*

1) Coombs CA, Murphy EL, Laros RK: "Factors associated with postpartum hemorrhage with vaginal birth." Obstet Gynecol 1991; 77:69–76.
2) Bais JM, Eskes M, Pel M, Bonsel GJ, Bleker OP. Postpartum haemorrhage in nulliparous women: incidence and risk factors in low and high risk women. A Dutch population-based cohort study on standard (> or = 500 ml) and severe (> or = 1000 ml) postpartum haemorrhage. Eur J Obstet Gynecol Reprod Biol. 2004 Aug 10;115(2):166-72. doi: 10.1016/j.ejogrb.2003.12.008. PMID: 15262350.
3) Yin X, Zhang D, Wang W, Xu Y. The Risk Factors of Postpartum Urinary Retention for Women by Vaginal Birth: A Systematic Review and Meta-Analysis. Int Urogynecol J. 2024 Jul 6. doi: 10.1007/s00192-024-05853-w. Epub ahead of print. PMID: 38970657.
4) Larsson P-G, Platz-Christensen J-J, Bergman B, et al. Advantage or disadvantage of episiotomy compared with spontaneous perineal laceration. Gynecol Obstet Invest. 1991;31(4):213–216.
5) Ghulmiyyah L, Sinno S, Mirza F, Finianos E, Nassar AH. Episiotomy: history, present and future - a review. J Matern Fetal Neonatal Med. 2022;35(7):1386-91
6) Ejegård H, Ryding EL, Sjogren B. Sexuality after delivery with episiotomy: a long-term follow-up. Gynecol Obstet Invest. 2008;66(1):1-7. doi: 10.1159/000113464. Epub 2008 Jan 17. PMID: 18204265.
7) He S, Jiang H, Qian X, Garner P. Women's experience of episiotomy: a qualitative study from China. BMJ Open. 2020;10(7):e033354.
8) Martinez-Galiano JM, Martinez-Vazquez S, Rodriguez-Almagro J, Hernandez-Martinez A. The magnitude of the problem of obstetric violence and its associated factors: A cross-sectional study. Women Birth. 2021;34(5):e526-e36.

**Pg 8, Line 12 needs a reference.**

Thank you for your comment. A reference has been added. Please see page 8 line 12: *“As described in Desplanches et al, studying episiotomy and OASI needs to take into account specific clinical contexts (20).*

20) Desplanches T, Szczepanski E, Cottenet J, Semama D, Quantin C, Sagot P. A novel classification for evaluating episiotomy practices: application to the Burgundy perinatal network. BMC Pregnancy Childbirth. 2019;19(1):300.

**Reviewer #3: Thanks for the revised manuscript and responses to my original review. The revised manuscript covers my original set of queries.**
